# Genetic interactions between planar cell polarity genes cause diverse neural tube defects in mice

**DOI:** 10.1242/dmm.016758

**Published:** 2014-08-15

**Authors:** Jennifer N. Murdoch, Christine Damrau, Anju Paudyal, Debora Bogani, Sara Wells, Nicholas D. E. Greene, Philip Stanier, Andrew J. Copp

**Affiliations:** 1 Centre for Biomedical Sciences, School of Biological Sciences, Royal Holloway University of London, Egham, TW20 0RD, UK.; 2 MRC Harwell, Harwell Science and Innovation Campus, Oxfordshire, OX11 0RD, UK.; 3 Newlife Birth Defects Research Centre, Institute of Child Health, University College London, 30 Guilford Street, London, WC1N 1EH, UK.

**Keywords:** Neural tube defects, Planar cell polarity, Genetic interactions, Craniorachischisis, Multiple heterozygosity

## Abstract

Neural tube defects (NTDs) are among the commonest and most severe forms of developmental defect, characterized by disruption of the early embryonic events of central nervous system formation. NTDs have long been known to exhibit a strong genetic dependence, yet the identity of the genetic determinants remains largely undiscovered. Initiation of neural tube closure is disrupted in mice homozygous for mutations in planar cell polarity (PCP) pathway genes, providing a strong link between NTDs and PCP signaling. Recently, missense gene variants have been identified in PCP genes in humans with NTDs, although the range of phenotypes is greater than in the mouse mutants. In addition, the sequence variants detected in affected humans are heterozygous, and can often be detected in unaffected individuals. It has been suggested that interactions between multiple heterozygous gene mutations cause the NTDs in humans. To determine the phenotypes produced in double heterozygotes, we bred mice with all three pairwise combinations of *Vangl2^Lp^*, *Scrib^Crc^* and *Celsr1^Crsh^* mutations, the most intensively studied PCP mutants. The majority of double-mutant embryos had open NTDs, with the range of phenotypes including anencephaly and spina bifida, therefore reflecting the defects observed in humans. Strikingly, even on a uniform genetic background, variability in the penetrance and severity of the mutant phenotypes was observed between the different double-heterozygote combinations. Phenotypically, *Celsr1^Crsh^*;*Vangl2^Lp^*;*Scrib^Crc^* triply heterozygous mutants were no more severe than doubly heterozygous or singly homozygous mutants. We propose that some of the variation between double-mutant phenotypes could be attributed to the nature of the protein disruption in each allele: whereas *Scrib^Crc^* is a null mutant and produces no Scrib protein, *Celsr1^Crsh^* and *Vangl2^Lp^* homozygotes both express mutant proteins, consistent with dominant effects. The variable outcomes of these genetic interactions are of direct relevance to human patients and emphasize the importance of performing comprehensive genetic screens in humans.

## INTRODUCTION

Central nervous system (CNS) defects account for around 10% of congenital malformations, and include some of the commonest causes of infant death and severe handicap (Dolk et al., 2010). Prominent among CNS defects are disorders of early neural tube development, which can have drastic consequences for the fetus and newborn infant. Exposure of the persistently open neural tube to the amniotic fluid environment leads to degeneration of the neural tissue, resulting in irreparable neurological damage by birth (Stiefel et al., 2007). Folic acid supplementation has been used to help protect against neural tube defects (NTDs), although this is not sufficient to prevent all cases (Copp et al., 2013). With such devastating and irreversible consequences, gaining an understanding of the genetic risk factors for NTDs is of prime importance because it could pave the way towards improved genetic counselling for parents, and identification of novel primary preventive therapies.

Defects of neural tube formation can be divided into disorders of primary and secondary neurulation (Copp et al., 2013). Primary neurulation is characterized by bending of the neural plate and closure of the neural tube in the dorsal midline. Failure of the onset of primary neurulation generates the most severe NTD, craniorachischisis, in which the majority of the neural tube remains open, from midbrain to low spine. Failure of closure specifically in the cranial region gives rise to anencephaly, whereas disturbance of spinal neural tube closure leads to open spina bifida (myelomeningocele). Secondary neurulation occurs in the low sacral and coccygeal regions and involves cavitation within a solid cell mass, the medullary cord, to generate the secondary neural tube. There is no neural tube closure at this axial level and so defects are of the closed type, often called ‘closed spinal dysraphism’; these include lipomyelomeningocele and disorders of the filum terminale.

Up to 70% of the risk of NTDs is probably caused by genetic factors (Leck, 1974), and yet the main predisposing genes for human NTDs are largely unknown. NTDs rarely present as multiple cases in families but exhibit a mainly sporadic pattern. Taken together with the relatively high prevalence of NTDs across the world, this is consistent with a multifactorial polygenic or oligogenic pattern of inheritance, together with an important role for non-genetic factors.

Mutations in over 240 mouse genes cause NTDs (Harris and Juriloff, 2007; Harris and Juriloff, 2010), indicating the complex genetic requirements for successful neurulation. Although most mutants exhibit exencephaly and/or open spina bifida, a small number develop craniorachischisis and it is striking that most of these genes encode proteins functionally related to the planar cell polarity (PCP) pathway, a non-canonical Wnt-frizzled (Fzd) signaling cascade (Wu et al., 2011).

Several of the mouse mutants with craniorachischisis have mutations that disrupt core components of the PCP pathway, including Celsr1, Vangl1, Vangl2, Fzd3, Fzd6, Dvl1, Dvl2 and Dvl3 (Kibar et al., 2001; Murdoch et al., 2001a; Hamblet et al., 2002; Curtin et al., 2003; Wang et al., 2006a; Wang et al., 2006b; Merte et al., 2010; Andre et al., 2012). Other craniorachischisis mutants exhibit PCP phenotypes (e.g. in the inner ear), although the biochemical role of the protein in PCP signaling is unclear (e.g. Scrib, Ptk7) (Murdoch et al., 2003; Lu et al., 2004; Paudyal et al., 2010; Savory et al., 2011).

**TRANSLATIONAL IMPACT**

**Clinical issue**

The genetic causes of human neural tube defects (NTDs) are largely undefined. NTDs, such as spina bifida and anencephaly, are among the most common and severe forms of birth defect, affecting around 1 in 1000 births worldwide. Studies in mice have identified a large number of single gene mutations that cause NTDs in homozygous mutants. However, screens for mutations in these genes in individuals with NTDs have generally given unconvincing results. Although many studies have found sequence variants, most of these variants are only present as heterozygous changes in humans, and can also be found in unaffected individuals. However, humans are likely to be heterozygous for sequence variants at two, three or more loci, which might then interact to cause the birth defect. The planar cell polarity (PCP) signaling pathway is a key molecular pathway that has been clearly demonstrated to cause NTDs when disrupted in mouse mutants. Individuals with NTDs have also been found to carry heterozygous mutations in some PCP genes, although the range of defects is greater than that observed in mice.

**Results**

In this study the authors tested the hypothesis that interactions between multiple heterozygous mutations in mice might recapitulate the range of NTD phenotypes seen in humans. Mouse lines carrying mutations that affect three different genes of the PCP signaling pathway (namely *Vangl2^Lp^*, *Scrib^Crc^* and *Celsr1^Crsh^*) were bred together to create all possible pairwise combinations of mutations. The majority of double-mutant embryos yielded NTDs, with a wide range of phenotypes that reflected the range of open defects observed in humans, including anencephaly and spina bifida. Strikingly, even when the mice were bred onto a uniform genetic background, the double mutants still showed a range of disease phenotypes. Triple-mutant embryos were also generated but had no more severe phenotypes than doubly heterozygous or singly homozygous mutants.

**Implications and future directions**

The variable outcomes of these genetic interactions are of direct relevance to the human condition. This study emphasizes the importance of performing comprehensive genetic screens in humans when searching for the genetic cause of birth defects. Although heterozygous mutation in a single gene might have no effect, disruption of multiple genes could combine in an individual to cause devastating consequences on embryonic development. The potential effect of genetic variants should not be underestimated even if detected in unaffected individuals, because the combination of multiple mutations is probably the critical factor in human NTDs.

In recent years, PCP genes have been strongly implicated as having a causal role in humans with NTDs (recently reviewed in Juriloff and Harris, 2012). Single-nucleotide variants, predominantly heterozygous non-synonymous (missense) genomic alterations, have been found in the coding region of the core PCP genes *CELSR1*, *FZD6*, *PRICKLE1*, *DVL2*, *VANGL1* and *VANGL2*, and in the PCP-associated genes *SEC24B*, *DACT1*, *FUZ* and *SCRIB* (Kibar et al., 2007; Kibar et al., 2009; Lei et al., 2010; Bosoi et al., 2011; Kibar et al., 2011; Seo et al., 2011; Allache et al., 2012; Bartsch et al., 2012; De Marco et al., 2012; Robinson et al., 2012; Shi et al., 2012; De Marco et al., 2013; Yang et al., 2013). In general, these ‘mutations’ are absent from, or very rare in, public databases such as the Single Nucleotide Polymorphism Database (dbSNP), the 1000 Genomes Project and the NHLBI Exome Variant Server. Typically, only a small proportion of the NTD cases in each study have a missense mutation and the variants, where studied, are inherited from a parent who lacks an NTD him/herself. Some but not all reports describe functional studies that demonstrate detrimental effects of the missense change on wild-type protein function, providing evidence for a disease-causing effect.

Strikingly, the NTDs and related phenotypes that are exhibited by individuals with putative PCP mutations vary widely, ranging from the open defects craniorachischisis, anencephaly and myelomeningocele, to the overt (but closed) defects of lipomyelomeningocele, terminal myelocystocele and sacral agenesis, and even include the internal disorders of diastematomyelia (split cord) and disorders of the filum terminale (Juriloff and Harris, 2012). The broad range of human PCP-associated overt NTDs contrasts with the typical association of mouse PCP mutations with craniorachischisis in homozygotes and only tail defects (or no overt spinal phenotype) in heterozygotes; studies to screen for internal disorders have not been performed. In humans, it is possible that the reported PCP variants act as dominant mutations with variable penetrance, although it has also been suggested that they might interact in a digenic or polygenic fashion with other, as yet unidentified, genetic NTD risk factors to generate the observed diversity of NTD types. Indeed, in mice, the core PCP gene *Vangl2* is known to interact with non-PCP genes to yield either open spina bifida or exencephaly (Greene et al., 2009), providing a paradigm for such gene interactions in causing variable NTD types.

The aim of the present study was to extend the analysis of PCP gene interactions by examining the range of open defects observed in pairwise or triply heterozygous combinations of PCP mutations. *Vangl2*, *Scrib* and *Celsr1* are among the most intensively studied mouse PCP genes, each of which causes craniorachischisis in homozygous mutants (Copp et al., 2003). Their human orthologs are all implicated in NTDs via missense mutations. We generated all pairwise combinations of the *Vangl2^Lp^*, *Scrib^Crc^* and *Celsr1^Crsh^* alleles, and observed a surprisingly wide range of NTD phenotypes both within individual crosses and between the different combinations of heterozygous mutations. Importantly, we show that this phenotypic variability remained even after breeding all three mutant lines onto the C3H/HeH strain for at least six generations to create a uniform genetic background, arguing against an effect of genetic modifiers. To begin to evaluate the mechanisms underlying these apparently diverse gene interactions, we determined the earliest disorder of neural tube closure in each mutant, and assessed the quantitative aspects of each PCP protein’s expression in all three PCP mutants. Our findings are consistent with both loss- and gain-of-function disorders among the PCP mutants, and suggest that the wide range of NTD-like phenotypes that are associated with human PCP mutations might be explained by the inheritance of different combinations of mutant alleles in different individuals.

## RESULTS

### Double PCP mutants develop craniorachischisis, with variable penetrance on a mixed genetic background

**Fig. 1.**
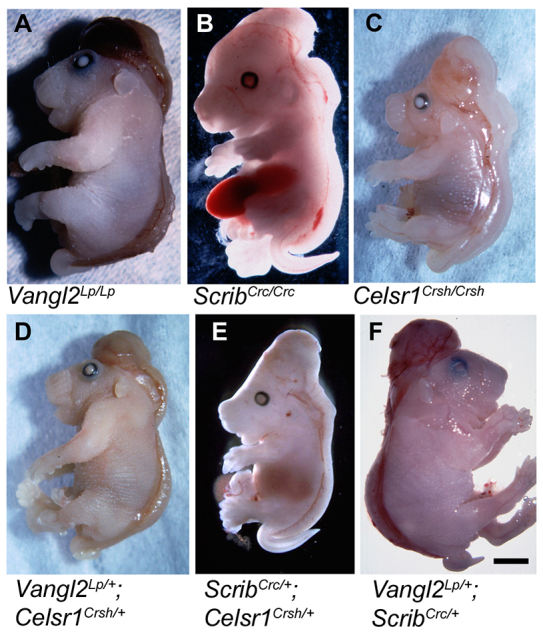
***Vangl2^Lp^*, *Scrib^Crc^* and *Celsr1^Crsh^* homozygous mutant embryos and doubly heterozygous mutants all exhibit craniorachischisis.** Lateral views of whole embryos, showing similar defects in the different genotype combinations. Embryos are either single-mutant homozygotes: (A) *Vangl2^Lp/Lp^*; (B) *Scrib^Crc/Crc^*; (C) *Celsr1^Crsh/Crsh^*; or double heterozygotes: (D) *Vangl2*^*Lp*/+^;*Celsr1*^*Crsh*/+^; (E) *Scrib*^*Crc*/+^;*Celsr1*^*Crsh*/+^; (F) *Vangl2*^*Lp*/+^;*Scrib*^*Crc*/+^. In addition to craniorachischisis, defects of ventral body wall closure (B) and eyelid closure (A–E) are present.

On a variable genetic background, homozygotes for each PCP gene – *Vangl2^Lp/Lp^*, *Scrib^Crc/Crc^* or *Celsr1^Crsh/Crsh^* – exhibit the severe neural tube closure defect craniorachischisis as a fully penetrant phenotype (Fig. 1A–C), as reported previously (Murdoch et al., 2001a; Murdoch et al., 2001b; Curtin et al., 2003). In contrast, although double heterozygotes also exhibited craniorachischisis (Fig. 1D–F), this occurred with incomplete penetrance on a mixed genetic background (Table 1A). *Vangl2*^*Lp*/+^;*Celsr1*^*Crsh*/+^ double heterozygotes developed craniorachischisis in 54% of cases (*n*=7/13) with the remainder exhibiting only a looped tail, closely similar to the 54% penetrance of craniorachischisis observed in *Vangl2*^*Lp*/+^;*Scrib*^*Crc*/+^ embryos (*n*=29/54) (Murdoch et al., 2001b). In contrast, *Scrib*^*Crc*/+^;*Celsr1*^*Crsh*/+^ mice exhibited only 8% penetrance of craniorachischisis (*n*=1/12).

**Table 1.**
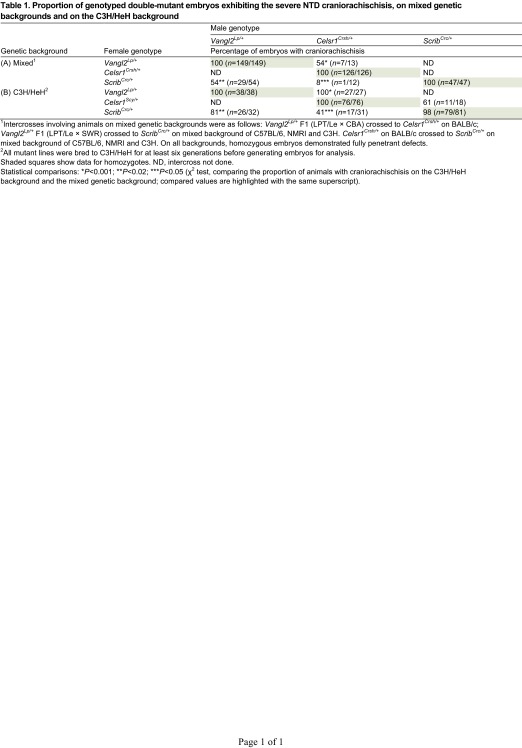
Proportion of genotyped double-mutant embryos exhibiting the severe NTD craniorachischisis, on mixed genetic backgrounds and on the C3H/HeH background

The significance of the varying craniorachischisis penetrance in these intercrosses was confounded by the different genetic background of each mutant (Table 1A). To overcome this limitation, we bred each mutant onto the same C3H/HeH genetic background, for at least six generations, to generate (sub-)congenic strains that, on average, carried C3H/HeH alleles in more than 98% of the genome.

### Genetic interactions between *Vangl2^Lp^*, *Scrib^Crc^* and *Celsr1^Crsh^* on the C3H/HeH genetic background show increased penetrance of craniorachischisis

Most genotype combinations were produced in the expected Mendelian ratios in the PCP intercrosses, except for the *Vangl2^Lp^* allele, which was inherited more frequently than the wild-type allele (supplementary material Table S1), as noted previously (Murdoch et al., 2001b). While generating the sub-congenic strains, heterozygous phenotypes (shaky-head behavior in *Celsr1*^*Crsh*/+^ and looped tail in *Vangl2*^*Lp*/+^) became less penetrant on the C3H/HeH background (supplementary material Table S2). Double heterozygotes between *Vangl2*^*Lp*/+^, *Scrib*^*Crc*/+^ and *Celsr1*^*Crsh*/+^ showed an increased penetrance of craniorachischisis at embryonic day (E)12.5–E16.5 on the C3H/HeH background (Table 1B). Nevertheless, despite the common genetic background, the penetrance of craniorachischisis varied between the different crosses.

### *Vangl2^Lp^*, *Scrib^Crc^* and *Celsr1^Crsh^* interactions generate a range of birth defects

On the C3H/HeH genetic background, the different PCP intercrosses produced a range of congenital defects with variable penetrance (Table 2).

**Table 2.**
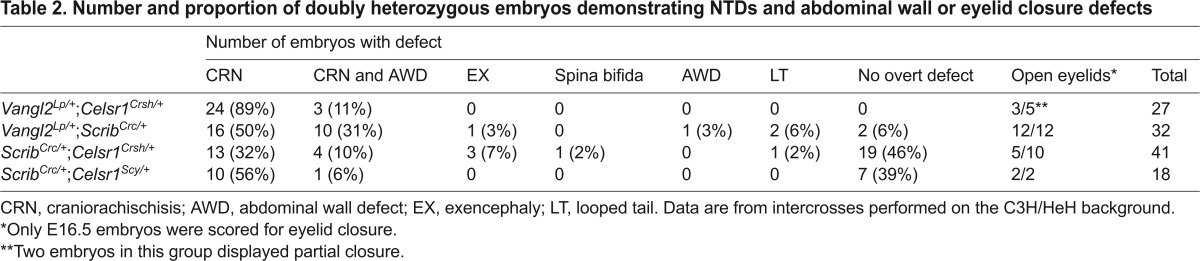
Number and proportion of doubly heterozygous embryos demonstrating NTDs and abdominal wall or eyelid closure defects

### *Vangl2*^*Lp*/+^ × *Celsr1*^*Crsh*/+^

**Fig. 2.**
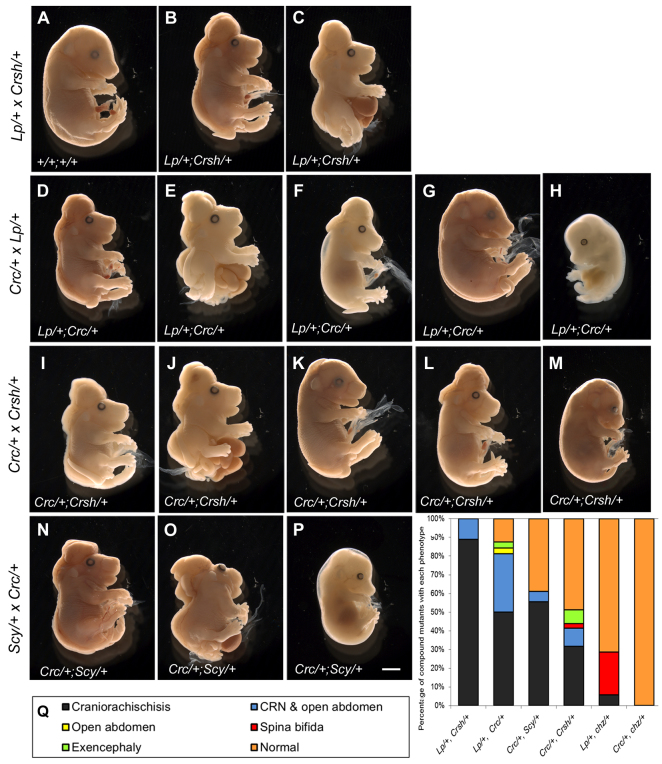
**PCP double heterozygotes exhibit a range of phenotypes.** Lateral views of embryos at E14.5–E16.5, illustrating the range of phenotypes observed in each cross. (A–C) *Vangl2*^*Lp*/+^ × *Celsr1*^*Crsh*/+^ cross (E16.5). Wild-type embryo (A) shows complete neural tube closure, with closed eyelids. Double heterozygotes (*Vangl2*^*Lp*/+^;*Celsr1*^*Crsh*/+^) have craniorachischisis without (B) or with (C) an abdominal wall defect, in which the liver and intestine protrude from the abdominal cavity (C). Both exhibit failed eyelid closure (B,C). (D–H) *Scrib*^*Crc*/+^ × *Vangl2*^*Lp*/+^ cross, at E16.5 (D,E,G), E15.5 (F) or E14.5 (H). The *Vangl2*^*Lp*/+^;*Scrib*^*Crc*/+^ double mutants exhibit isolated craniorachischisis (D), craniorachischisis and abdominal wall defect (E), exencephaly and a looped tail (F), closed neural tube but looped tail (G), or complete neural tube closure but abdominal wall defect with protruding liver (H). At E16.5, some double-mutant embryos exhibit failure of eyelid closure (D–F), whereas others have closed eyelids (G). (I–M) Embryos from *Scrib*^*Crc*/+^ × *Celsr1*^*Crsh*/+^ cross, at E16.5 (I–K) or E15.5 (L,M). *Scrib*^*Crc*/+^;*Celsr1*^*Crsh*/+^ double mutants exhibit isolated craniorachischisis (I), craniorachischisis and abdominal wall defect with protruding abdominal contents (J), lumbosacral spina bifida (K), isolated exencephaly (L), or appear morphologically normal (M). Both open (I,J) and closed (K) eyelids are observed. (N–P) Embryos from *Celsr1*^*Scy*/+^ × *Scrib*^*Crc*/+^ cross at E16.5 (N,O) or E15.5 (P). *Scrib*^*Crc*/+^;*Celsr1*^*Scy*/+^ double mutants exhibit isolated craniorachischisis (N), or craniorachischisis with abdominal wall defect (O), both with failure of eyelid closure. Other *Scrib*^*Crc*/+^;*Celsr1*^*Scy*/+^ double mutants are overtly normal (P). (Q) Range and proportion of phenotypes observed in different doubly heterozygous mutants, on the C3H/HeH background. Phenotypes are craniorachischisis (black), craniorachischisis and abdominal wall defect (blue), abdominal wall defect without craniorachischisis (yellow), spina bifida (red), exencephaly (green) or no overt defect (orange). Data combined from the present study and from our previous work on *Ptk7^chz^* mutant interactions (Paudyal et al., 2010). Scale bar: 2 mm.

Craniorachischisis occurred in 100% of doubly heterozygous embryos (*n*=27), usually as an isolated defect (*n*=24/27; Fig. 2A,B) but sometimes associated with an abdominal wall defect (Fig. 2C; *n*=3/27), which is likely to be omphalocele/exomphalos (Carnaghan et al., 2013). The abdominal wall defect is seen in both *Vangl2^Lp/Lp^* and *Celsr1^Crsh/Crsh^* mutants, at low incidence (<5% and ~10%, respectively). The abdominal wall defect coincided with a rightward skewing of the embryo, with obvious shortening of the right-hand side compared with the left (Fig. 2C), suggesting a defect in embryonic turning. Some *Vangl2*^*Lp*/+^;*Celsr1*^*Crsh*/+^ double mutants collected at E16.5 had failed to close their eyelids (Fig. 2B,C; *n*=3/5), as seen also in *Vangl2^Lp/Lp^* and *Celsr1^Crsh/Crsh^* homozygous mutants (Curtin et al., 2003), whereas others showed partial eyelid closure (*n*=2/5).

### *Vangl2*^*Lp*/+^ × *Scrib*^*Crc*/+^

Craniorachischisis was also observed in the majority of doubly heterozygous fetuses (Fig. 2D,E; *n*=26/32; 81%), most often isolated (*n*=16/26; Fig. 2D), but sometimes associated with abdominal wall defect and a skewed body axis (*n*=10/26; Fig. 2E). One *Vangl2*^*Lp*/+^;*Scrib*^*Crc*/+^ fetus exhibited abdominal wall defect with a closed neural tube (Fig. 2H). Other double mutants exhibited exencephaly (*n*=1/32; 3%; Fig. 2F) or had only a tail defect (Fig. 2G; *n*=2/32) or no overt defect (*n*=2/32). Double heterozygotes examined at E16.5 exhibited failure of eyelid closure (Fig. 2D,E; *n*=12/12); all of these fetuses also demonstrated craniorachischisis.

### *Scrib*^*Crc*/+^ × *Celsr1*^*Crsh*/+^

This cross resulted in craniorachischisis in 42% of doubly heterozygous fetuses (*n*=17/41), most often in isolation (*n*=13/17; Fig. 2I), but sometimes associated with abdominal wall defect and a skewed body axis (*n*=4/17; Fig. 2J). Other defects seen in *Scrib*^*Crc*/+^;*Celsr1*^*Crsh*/+^ fetuses included lumbosacral open spina bifida (2%, *n*=1/41; Fig. 2K) or isolated exencephaly (7%, *n*=3/41; Fig. 2L), although many appeared phenotypically normal (46%, *n*=19/41; Fig. 2M) or had only a tail defect (2%, *n*=1/41). Some *Scrib*^*Crc*/+^;*Celsr1*^*Crsh*/+^ mutants exhibited failure of eyelid formation at E16.5 (Fig. 2I,J; *n*=5/10); those mutants with craniorachischisis showed completely open eyelids, whereas the one individual with open spina bifida exhibited partially closed eyelids (Fig. 2K), and those with normal neural tube closure displayed closed eyelids.

A cross between *Scrib*^*Crc*/+^ and a second *Celsr1* mutant allele, *spin-cycle* (Curtin et al., 2003) also generated doubly heterozygous fetuses with isolated craniorachischisis (*n*=10/18; Fig. 2N), or craniorachischisis with abdominal wall defect (*n*=1/18; Fig. 2O), as well as eyelid closure defects (*n*=2/2; Fig. 2N,O). A proportion of *Scrib*^*Crc*/+^;*Celsr1*^*Scy*/+^ fetuses were overtly normal (Fig. 2P; *n*=7/18).

Hence, although breeding *Vangl2^Lp^*, *Scrib^Crc^* and *Celsr1^Crsh^* onto the C3H/HeH background increases the penetrance of craniorachischisis in double heterozygotes, there remains a considerable difference in defect penetrance between the different intercrosses. The variability is even more dramatic when considered together with our previous findings with the *Ptk7* mutant allele *chuzhoi* (Paudyal et al., 2010). Craniorachischisis occurred at very low penetrance in *Vangl2*^*Lp*/+^;*Ptk7*^*chz*/+^ double heterozygotes and was not observed at all in *Scrib*^*Crc*/+^;*Ptk7*^*chz*/+^ or *Celsr1*^*Crsh*/+^;*Ptk7*^*chz*/+^ double mutants, on a C3H/HeH background. Isolated open spina bifida was the more commonly observed NTD in these crosses (Paudyal et al., 2010). The graph in Fig. 2 summarizes the range of phenotypes and their penetrance in this and the previous study (Paudyal et al., 2010).

### Phenotype of triply heterozygous *Vangl2*^*Lp*/+^;*Scrib*^*Crc*/+^;*Celsr1*^*Crsh*/+^ mutant embryos

**Fig. 3.**
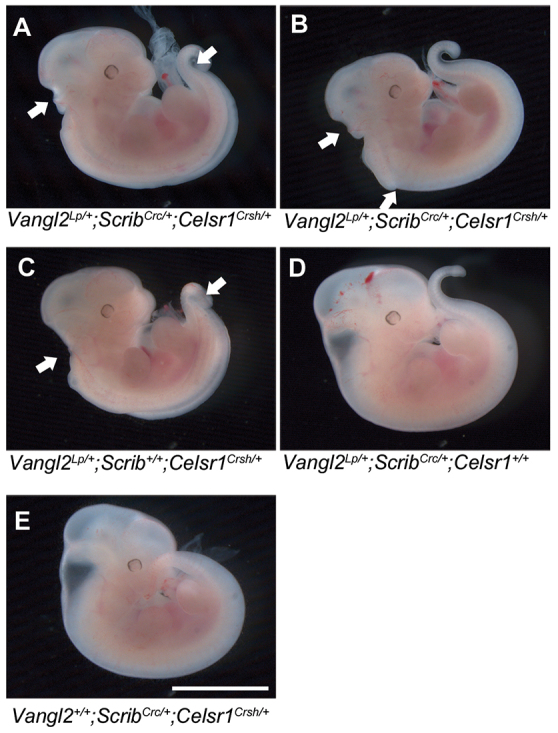
**Triply heterozygous PCP mutant embryos have no more severe phenotypes than double heterozygotes.** Embryos at E10.5 generated from intercross of a *Celsr1*^*Crsh*/+^ female with a *Vangl2*^*Lp*/+^;*Scrib*^*Crc*/+^ male. (A) Triple mutant *Vangl2*^*Lp*/+^;*Scrib*^*Crc*/+^;*Celsr1*^*Crsh*/+^ with craniorachischisis. (B) Triple mutant *Vangl2*^*Lp*/+^;*Scrib*^*Crc*/+^;*Celsr1*^*Crsh*/+^ with hindbrain exencephaly. (C) Double mutant *Vangl2*^*Lp*/+^;*Scrib*^+^*^/^*^+^;*Celsr1*^*Crsh*/+^ with craniorachischisis. (D) Double mutant *Vangl2*^*Lp*/+^;*Scrib*^*Crc*/+^;*Celsr1*^+/+^ with a looped-tail phenotype. (E) Double mutant *Vangl2*^+/+^;*Scrib*^*Crc*/+^;*Celsr1*^*Crsh*/+^ with no overt phenotype. Arrows in A, B and C indicate the cranial and caudal ends of the open region, in each embryo. Scale bar: 1 mm.

While analyzing the double PCP mutants, we recovered some postnatally viable *Vangl2*^*Lp*/+^;*Scrib*^*Crc*/+^ double heterozygotes (13%), albeit at a lower proportion than observed previously (~50%) (Murdoch et al., 2001b). Surviving *Vangl2*^*Lp*/+^;*Scrib*^*Crc*/+^ double-mutant males were bred with *Celsr1*^*Crsh*/+^ females to generate triple-mutant embryos. Despite considerably reduced fertility in this cross, all genotype combinations were observed, and three triply heterozygous *Vangl2*^*Lp*/+^;*Scrib*^*Crc*/+^;*Celsr1*^*Crsh*/+^ embryos were obtained (3/22; 14%), close to the 1 in 8 expectation. Of these, two exhibited craniorachischisis (example shown in Fig. 3A), whereas the third had hindbrain exencephaly and a tail defect (Fig. 3B; supplementary material Table S3). These findings suggest that triple heterozygosity for the three mutant PCP loci is no more severe than any of the double mutants (Fig. 3C–E).

### Delay in initiation of neural tube closure varies between PCP heterozygotes

**Fig. 4.**
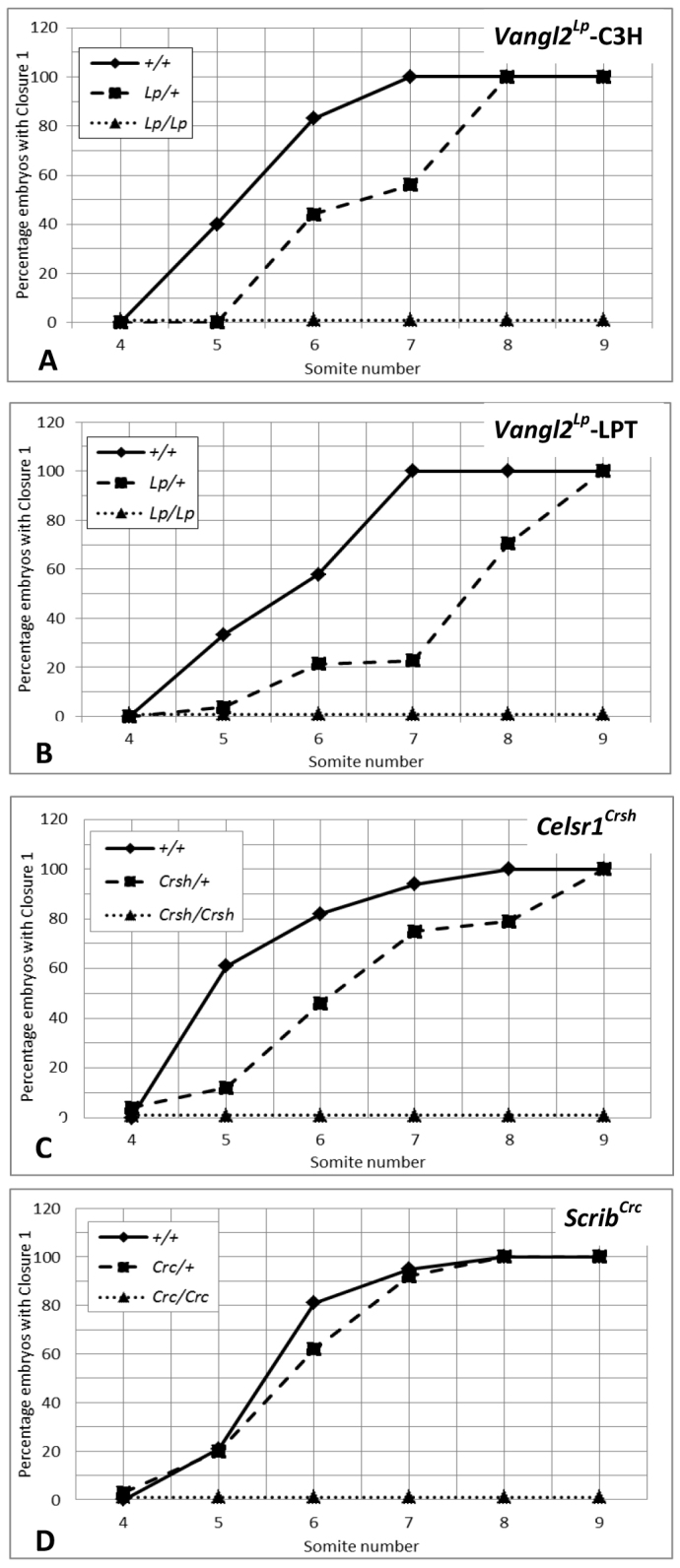
**Delayed initiation of neural tube closure in *Celsr1*^*Crsh*/+^ and *Vangl2*^*Lp*/+^ but not in *Scrib*^*Crc*/+^ embryos.** Proportion of E8.5 embryos that initiate neural tube closure in the four- to nine-somite stage interval is shown for wild type (solid line), heterozygous mutants (dashed line) and homozygous mutants (dotted line). (A) *Vangl2^Lp^* mutant strain (congenic on C3H/HeH; *n*=22 +/+, 42 *Lp*/+, 16 *Lp/Lp*); (B) *Vangl2^Lp^* mutant strain (on LPT/Le × CBA background; *n*=55 +/+, 131 *Lp*/+, 49 *Lp/Lp*); (C) *Celsr1^Crsh^* mutant strain (*n*=75 +/+, 175 *Crsh*/+, 61 *Crsh/Crsh*); (D) *Scrib^Crc^* mutant strain (congenic on C3H/HeH; *n*=163 +/+, 371 *Crc*/+, 154 *Crc/Crc*). Homozygotes all fail to initiate neural tube closure. Wild-type embryos undergo Closure 1 between the five- and eight-somite stages. *Vangl2*^*Lp*/+^ and *Celsr1*^*Crsh*/+^ heterozygotes are delayed in closure initiation compared with wild type (*Vangl2^Lp^-C3H*: *P*=0.03; *Vangl2^Lp^-LPT*: *P*<0.001; *Celsr1^Crsh^*: *P*<0.001), whereas *Scrib*^*Crc*/+^ embryos do not differ from wild-type littermates (*P*=0.49).

We showed previously that *Vangl2^Lp^* homozygotes develop craniorachischisis owing to failure of initiation of neural tube closure at the six-somite stage (Copp et al., 1994). Moreover, *Vangl2*^*Lp*/+^ embryos are delayed in closure initiation, although ultimately all heterozygotes achieve Closure 1 (Copp et al., 1994). *Vangl2^Lp/Lp^* embryos on the C3H/He background also failed in Closure 1, although the closure delay in *Vangl2*^*Lp*/+^ was less severe (1.2-somites delay; Fig. 4A) than on the original LPT/CBA background (1.9-somites delay; Fig. 4B).

We investigated whether *Celsr1^Crsh^* and *Scrib^Crc^* embryos have similar early developmental defects as *Vangl2^Lp^*. In *Celsr1^Crsh^* litters (Fig. 4C), wild-type embryos achieved closure between the five- and eight-somite stages (*n*=75 wild-type embryos). In contrast, *Celsr1^Crsh/Crsh^* embryos failed to initiate closure, with 100% open neural tubes even at the nine-somite stage. *Celsr1*^*Crsh*/+^ heterozygotes exhibited delayed closure, of ~1.3 somite stages (4.8 to 6.1), relative to wild-type littermates (Fig. 4C). Nevertheless, all *Celsr1*^*Crsh*/+^ embryos initiated closure by the nine-somite stage.

Analysis of E8.5 *Scrib^Crc^* litters produced a rather different result (Fig. 4D). Wild-type embryos achieved Closure 1 between the five-and eight-somite stages, whereas all *Scrib* homozygotes failed in Closure 1. Notably, however, *Scrib^Crc^* heterozygotes initiated closure with a closely similar pattern to wild-type littermates. We conclude that craniorachischisis originates through failure of Closure 1 in homozygotes for all three PCP mutants, but that *Scrib*^*Crc*/+^ embryos do not exhibit delayed closure, unlike *Celsr1* or *Vangl2* heterozygotes.

### Abnormal neural plate morphology in *Celsr1^Crsh/Crsh^* mutants

The failure to initiate neural tube closure in *Vangl2^Lp/Lp^* and *Scrib^Crc/Crc^* mutants has been attributed to abnormal morphology of the neural plate, prior to the stage of Closure 1. The neural plate midline is broadened, with a disrupted median hinge point that mechanically prevents the neural tube from closing (Greene et al., 1998; Murdoch et al., 2003). We compared homozygous *Celsr1^Crsh/Crsh^* mutants with wild-type littermates around the time of closure initiation and found a broader neural plate with enlargement of the ventral midline and notochord (supplementary material Fig. S1A,B). The midline markers *Shh* and *Foxa2* exhibited a broadened expression domain, with bifurcation of *Foxa2* expression along part of the embryonic axis (supplementary material Fig. S1C–J,O,P), similar to *Vangl2^Lp^* mutants (Greene et al., 1998). *Vangl2* expression was largely unaltered in *Celsr1^Crsh/Crsh^* homozygotes (supplementary material Fig. S1K,L), although the neural plate was broader overall and the region lacking *Vangl2* expression in the ventral midline was enlarged (supplementary material Fig. S1M,N,Q,R). We conclude that craniorachischisis in all three PCP mutants arises from a common embryonic defect, affecting the neural plate midline.

### Protein expression analysis suggests that *Scrib^Crc^* is a null mutant, whereas *Vangl2^Lp^* and *Celsr1^Crsh^* express mutant protein isoforms

**Fig. 5.**
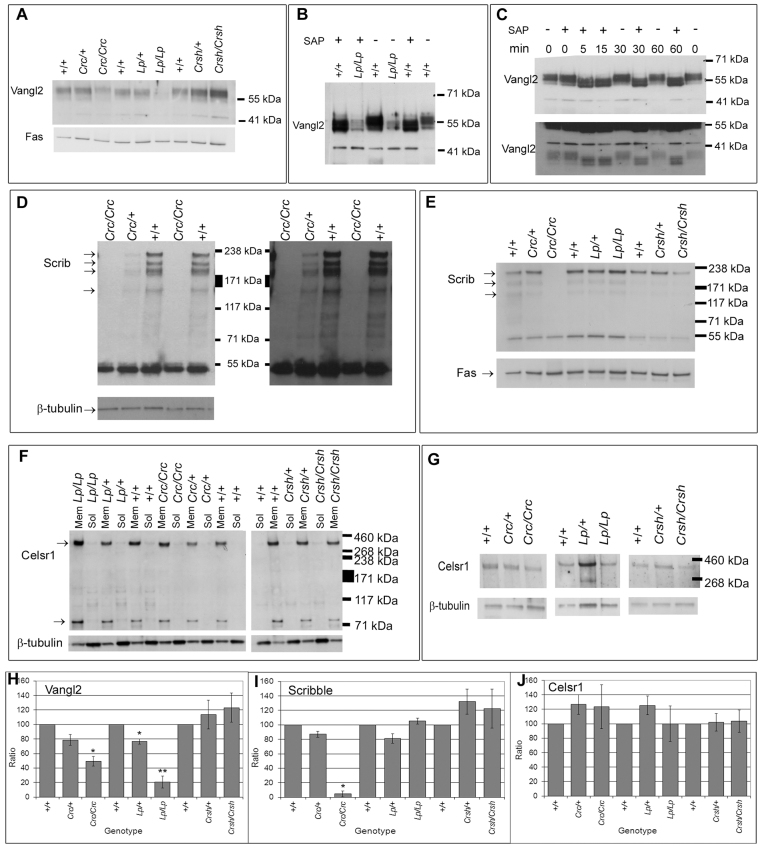
**Protein analysis suggests that *Scrib^Crc^* is a null mutant, whereas *Vangl2^Lp^* and *Celsr1^Crsh^* express mutant protein isoforms.** Western blot analysis was performed on whole embryo lysates (unless otherwise stated) using antibodies specific for the individual PCP proteins. (A) Vangl2 protein expression in E8.5 wild-type, heterozygous and homozygous embryos for *Scrib^Crc^*, *Vangl2^Lp^* and *Celsr1^Crsh^*. Vangl2 has reduced abundance in *Vangl2^Lp/Lp^* and to a lesser extent in *Scrib^Crc^* embryos. Fatty acid synthase (Fas) was used as loading control. (B) Vangl2 protein expression in membrane fractions from E10.5 wild-type and *Vangl2^Lp/Lp^* embryos, following incubation with (+) or without (−) shrimp alkaline phosphatase (SAP). *Vangl2^Lp/Lp^* mutants show reduced Vangl2 expression, and SAP treatment causes a shift in band sizes in wild type consistent with decreased phosphorylation in *Vangl2^Lp/Lp^* mutants. (C) Vangl2 protein expression in wild-type E11.5 membrane and organelle fraction, treated with (+) or without (−) SAP, for times from 0 to 60 minutes as indicated above each lane. SAP incubation causes a shift in band distribution to a lower molecular weight, although three bands can still be seen (upper panel). This suggests that Vangl2 is phosphorylated endogenously, but that other modifications are also present, giving rise to the multiple bands observed. Extended exposure reveals bands at lower molecular weights (lower panel), indicating the existence of shorter isoforms (or cleavage products), that might correspond to alternative splice variants predicted on Ensembl. (D) Scrib protein expression in membrane fractions of E13.5 wild-type, *Scrib*^*Crc*/+^ and *Scrib^Crc/Crc^* fetuses. A duplicate blot was immunostained for β-tubulin. Scrib is detected as multiple isoforms from ~210 kDa (arrows). *Scrib^Crc/Crc^* mutants completely lack Scrib protein, with no evidence for stably expressed truncated product, even after extended exposures (right-hand panel). The monoclonal antibody detects a band at ~55 kDa, in both wild-type and *Scrib^Crc/Crc^* mutants; no known splice variants are predicted to generate a shorter isoform of this size, and this band seems likely the result of nonspecific antibody binding. (E) Scrib expression in E8.5 wild-type, heterozygous and homozygous embryos from *Scrib^Crc^*, *Vangl2^Lp^* and *Celsr1^Crsh^* litters. Scrib is absent from *Scrib^Crc/Crc^* embryos. The lower molecular weight bands varied in intensity between protein preparations and could be either degradation products of Scrib protein or alternatively spliced isoforms. (F,G) Celsr1 protein expression in membrane (Mem) and soluble (Sol) fractions of E10.5 embryos (F) and whole-cell lysates of E8.5 embryos (G) from *Vangl2^Lp^*, *Scrib^Crc^* and *Celsr1^Crsh^* litters. Celsr1 bands (arrows in F) are detected at ~350 kDa (F,G) and ~80 kDa (F), with no apparent change in any mutants. (H–J) Quantitation of Vangl2 (H), Scrib (I) and Celsr1 (J) protein expression in E8.5 *Scrib^Crc^*, *Vangl2^Lp^* and *Celsr1^Crsh^* embryos, normalized to β-tubulin (H) or Fas (I,J) levels. Data are the average of three experiments ± standard errors; expression levels that differ significantly from wild type are indicated with asterisks (*P<0.05, **P<0.01).

We analyzed protein abundance by western blotting to determine whether the *Vangl2^Lp^*, *Scrib^Crc^* and *Celsr1^Crsh^* mutations result in altered expression or stability of their respective proteins. We confirmed evidence from others suggesting that *Vangl2^Lp^* and *Celsr1^Crsh^* express mutant proteins that can potentially have dominant effects. As reported previously (Gao et al., 2011; Belotti et al., 2012; Yin et al., 2012), Vangl2 protein remained detectable in *Vangl2^Lp/Lp^* mutants although with a marked (~80%) reduction in abundance and downward shift in molecular mass that is emulated by dephosphorylation of wild-type protein (Fig. 5A–C,H); we note that multiple bands remained after dephosphorylation, suggesting additional causes of size variants. Celsr1 showed no difference in size or abundance in *Celsr1^Crsh/Crsh^* mutants compared with wild-type littermates (Fig. 5F,G,J), consistent with previous reports (Devenport and Fuchs, 2008), despite carrying an amino acid substitution within the extracellular domain (Curtin et al., 2003).

*Scrib^Crc^* carries a single-base insertion that is predicted to cause a frameshift and premature truncation of the Scrib protein (Murdoch et al., 2003). In theory, this truncated isoform (971 amino acids; 100–120 kDa) could be stably expressed and, in fact, alternative splicing generates a similar isoform in *Drosophila* (Li et al., 2001). Others did not detect Scrib protein in *Scrib^Crc^* mutants (Moreau et al., 2010) but their antibodies recognized the C-terminal region of Scrib and could not detect a truncated isoform. We used a mouse monoclonal antibody specific for the Scrib N-terminal domain (Dow et al., 2003), to detect both full-length and truncated proteins. Wild-type embryos revealed multiple bands, the largest (~210 kDa) corresponding to full-length Scrib protein (Fig. 5D). *Scrib^Crc/Crc^* homozygous mutants showed a complete absence of the full-length Scrib protein (Fig. 5D,E,I), whereas *Scrib*^*Crc*/+^ heterozygotes demonstrated substantially reduced Scrib expression. Extended exposures provided no evidence for a truncated, 100–120 kDa, isoform in *Scrib^Crc/Crc^* mutants (Fig. 5D, right panel). Therefore, the evidence suggests that *Scrib^Crc^* is a complete loss-of-function mutant.

### PCP protein expression in *Vangl2^Lp^*, *Scrib^Crc^* and *Celsr1^Crsh^* single mutants

Western blot analysis showed that *Scrib^Crc/Crc^* mutants exhibited a moderate (~50%) reduction in Vangl2 abundance, compared with wild-type littermates, whereas *Celsr1^Crsh/Crsh^* mutants had no detectable change in Vangl2 expression (Fig. 5A,H). Scrib protein abundance was unaffected in E8.5 *Vangl2^Lp^* or *Celsr1^Crsh^* homozygous or heterozygous mutants (Fig. 5E,I). Similarly, the abundance of Celsr1 protein was unchanged in *Vangl2^Lp/Lp^* and *Scrib^Crc/Crc^* single-mutant embryos at E10.5 and at E8.5 (Fig. 5F,G,J).

## DISCUSSION

Recent human genetic studies have identified putative PCP mutations associated with a variety of NTDs and related phenotypes (Juriloff and Harris, 2012). Among the defects found in individuals with rare missense PCP gene mutations are craniorachischisis, other open NTDs (e.g. anencephaly, myelomeningocele), closed NTDs (e.g. lipomyelomeningocele) and gastrulation defects (e.g. sacral agenesis). This challenges the view that disruption of PCP gives rise specifically to craniorachischisis whereas other NTDs, such as anencephaly and spina bifida, are likely to result from different cellular or molecular defects (Copp et al., 2003).

The present study addressed this issue experimentally by examining the doubly heterozygous phenotypes of pairs of PCP gene mutations, for which craniorachischisis is the only homozygous phenotype. The result is unequivocal: craniorachischisis and other open NTDs, both exencephaly and open spina bifida (myelomeningocele), are all possible outcomes of the same PCP gene interaction. Some double heterozygotes exhibited tail flexion defects, which might result from a disorder of secondary neurulation (Copp et al., 1982); indeed, putative human PCP mutations are associated with disorders of secondary neurulation. Importantly, a proportion of the doubly heterozygous mice had no discernible phenotype, perhaps paralleling the unaffected parents who were found to transmit a missense PCP mutation to their offspring.

It is important to note that human patients with missense PCP mutations were single heterozygotes, and it is generally assumed that heterozygosity for one or more additional deleterious interacting mutations must be present (Juriloff and Harris, 2012). The current study enhances the plausibility of this hypothesis, although the ‘second mutation’ has yet to be identified in the majority of human cases. Three out of five human fetuses with craniorachischisis were found to have two rare variants of *CELSR1* (Robinson et al., 2012) and three individuals with lipomyelomeningocele or spinal lipoma each had two rare variants of *CELSR1* (Allache et al., 2012). To date, only four cases of double heterozygosity for mutations in two different PCP genes have been documented (De Marco et al., 2013). This might reflect the relatively small number of cases and the fact that the majority of studies have focused on individual candidate genes.

### Variations in NTD penetrance and expressivity – what are the possible causes?

We bred double heterozygotes for three prominent PCP gene mutations – *Celsr1^Crsh^*, *Vangl2^Lp^* and *Scrib^Crc^* – to study gene interactions in generating NTDs and to evaluate possible effects of modifiers within the genetic background. The starting point was the observation that all three homozygotes: *Celsr1^Crsh/Crsh^*, *Vangl2^Lp/Lp^* and *Scrib^Crc/Crc^*, exhibit 100% penetrance of craniorachischisis. Hence, all three genes are indispensable for initiation of neural tube closure. Despite this, each PCP intercross generated a range of phenotypes, both NTDs and defects not associated with the neural tube, with variable penetrance between gene combinations. Importantly, these phenotypic and penetrance differences persisted, even after minimizing genetic variability by breeding all three mutants to the same C3H/HeH background. This observation of retained variability despite the almost uniform genetic background argues strongly against the hypothesis of genetic modifier loci as the cause of the differences in the penetrance and types of NTDs observed between the different gene combinations. Therefore, several alternative reasons for this persistence of phenotypic differences can be considered.

### Differences in pathogenicity of the PCP mutants

It is possible that the three PCP mutant proteins cause varying degrees of developmental disturbance, leading to differences in penetrance and expressivity between the different double-heterozygote combinations. We and others detected Vangl2 protein in *Vangl2^Lp/Lp^* mutants, albeit at markedly reduced levels and with altered phosphorylation; however, the presence of this protein still indicates that the mutant protein might exert a dominant effect (Gao et al., 2011; Belotti et al., 2012; Yin et al., 2012). Indeed, Vangl2^Lp^ protein affects Vangl1 function (Belotti et al., 2012; Yin et al., 2012) and syndecan-4 protein stability (Escobedo et al., 2013), and the *Vangl2*^*Lp*/−^ compound mutant has a more severe phenotype than the *Vangl2*^−/−^ knockout (Yin et al., 2012). Similarly, we and others have found that mutant Celsr1^Crsh^ protein is expressed at normal levels, albeit with a subtly altered tissue distribution (Devenport and Fuchs, 2008; Formstone et al., 2010). *Celsr1^Crsh^* mice have a more severe phenotype than a *Celsr1* knockout null allele (Ravni et al., 2009; Qu et al., 2010), encouraging the view that Celsr1^Crsh^ mutant protein might also exert a dominant effect, for example by binding interactors and blocking their function.

In contrast to *Vangl2^Lp^* and *Celsr1^Crsh^*, we found evidence that the *Scrib^Crc^* mutation results in loss of Scrib protein. Although the *Scrib^Crc^* mutation is predicted to cause a frameshift and premature truncation (Murdoch et al., 2003), we could not detect a stably expressed truncated isoform. This suggests that the *Scrib*^*Crc*/+^ phenotype is due to haploinsufficiency, rather than dominant effects. Consistent with this idea, *Scrib^Crc^* heterozygotes exhibited normal initiation of neural tube closure, in contrast to *Celsr1*^*Crsh*/+^ and *Vangl2*^*Lp*/+^ embryos, which were delayed in closure initiation. Moreover, *Celsr1*^*Crsh*/+^;*Vangl2*^*Lp*/+^ double mutants had a more severe phenotype than either *Celsr1*^*Crsh*/+^;*Scrib*^*Crc*/+^ or *Vangl2*^*Lp*/+^;*Scrib*^*Crc*/+^. We hypothesize that the mutant alleles of *Celsr1^Crsh^* and *Vangl2^Lp^* lead to greater disruption of the pathway than the *Scrib^Crc^* mutation, explaining the greater severity of phenotypes in crosses involving the *Celsr1^Crsh^* and *Vangl2^Lp^* alleles.

Evidence from the *Ptk7^Chz^* mutant allele extends this hypothesis further. *Ptk7^Chz^* carries a three-amino-acid insertion in the extracellular domain of Ptk7, causing reduced abundance of the smaller protein isoform and decreased membrane localization (Paudyal et al., 2010). The *Ptk7^Chz^* mutation introduces an additional cleavage site for membrane type I matrix metalloproteinase, leading to instability of the cleaved ectodomain fragment (Golubkov et al., 2011). Therefore, *Ptk7^Chz^* is likely a hypomorphic allele, consistent with the finding that the *Ptk7* gene-trap allele (Lu et al., 2004) has a more severe phenotype than *Ptk7^Chz^*. Hence, double heterozygotes involving *Ptk7^Chz^* should have a lower penetrance of defects even than *Scrib^Crc^*, as observed.

### Differences in requirement of Celsr1, Vangl2 and Scrib for PCP signaling

Celsr1 and Vangl2 are considered core components of the PCP pathway, whereas Scrib is a more peripheral component, with known functions in other systems, including apico-basal polarity (Martin-Belmonte and Mostov, 2008). *Celsr1^Crsh/Crsh^* and *Vangl2^Lp/Lp^* both show loss of anterior-posterior alignment of hair follicles in skin, whereas no follicular defect is seen in *Scrib^Crc/Crc^* (Devenport and Fuchs, 2008). Ptk7 might also affect PCP indirectly; the planar-polarized localization of Dvl in mammalian cochlear sensory hair cells depends on the core PCP components Vangl2 and Fzd, but is independent of Ptk7 (Lee et al., 2012). This difference in requirement for each of the genes for PCP signaling correlates with the increased severity and penetrance of defects in double heterozygotes involving two core proteins (Vangl2 and Celsr1) compared with those involving one core and one more peripheral protein (Celsr1 and Scrib), or between two peripheral proteins (Scrib and Ptk7) (Paudyal et al., 2010). However, this hypothesis might also predict that *Ptk7*;*Vangl2* double heterozygotes should have a higher craniorachischisis frequency than *Ptk7* homozygotes, whereas we and others have observed the opposite (Lu et al., 2004; Paudyal et al., 2010).

### A role of persisting polymorphic modifier genes?

*Scrib^Crc^* and *Ptk7^chz^* (Paudyal et al., 2010) heterozygotes were selected for breeding solely by genotype, so the likelihood of retained modifiers is negligible. In contrast, mice were selected based on the looped tail phenotype for *Vangl2*^*Lp*/+^ and shaky-head behavior for *Celsr1*^*Crsh*/+^, making it possible that genetic modifiers of these phenotypes have been retained, in addition to the causative mutation. However, we observed a decline in the penetrance of heterozygous phenotypes after breeding to C3H/HeH, which can be argued as demonstrating the gradual loss of genetic variation at modifier loci. Moreover, because the frequency of craniorachischisis increased for all genotype combinations on the C3H/HeH background, one would have to argue that different modifier loci affect craniorachischisis versus heterozygous phenotypes. Therefore, the possibility of selective retention of modifiers is highly unlikely. Although the highly variable genetic background of human patients is likely to make a significant contribution to variation of the NTD phenotypes observed, this might not be the only factor involved. We demonstrate in our mouse studies that phenotypic variation still occurs even on a uniform genetic background.

### Comparison of double- and triple-heterozygote phenotypes

The most severe phenotype observed in the singly homozygous or any combination of doubly heterozygous PCP mutants was craniorachischisis, and the triply heterozygous embryos had phenotypes that were no more severe than in double heterozygotes. In genetic terms, it would seem that *Vangl2^Lp^* and *Celsr1^Crsh^* are epistatic to *Scrib^Crc^*, because the addition of a *Scrib^Crc^* mutation makes little difference to the phenotype of the *Vangl2*^*Lp*/+^;*Celsr1*^*Crsh*/+^ double mutant. Although craniorachischisis is a severe defect with devastating consequences for the embryo, it is perhaps not the exacerbation of the phenotype that might be expected from further disruption of the PCP pathway. Rather, because PCP regulates convergent extension (Keller et al., 2008), one might expect complete disruption of the pathway to impact on gastrulation, generating a more severe morphological defect and early embryo lethality. Indeed, mutants involving all three mammalian *dishevelled* genes exhibit developmental arrest at gastrulation stages (Wynshaw-Boris, 2012), although the phenotype could have been influenced by effects on the Wnt–β-catenin and Wnt-Ca^2+^ pathways, for which dishevelled proteins are also essential. It is possible that PCP pathway function is not completely ablated in either singly homozygous or doubly or triply heterozygous mutants for *Vangl2^Lp^*, *Celsr1^Crsh^* and *Scrib^Crc^*, and that craniorachischisis is the phenotype associated with partial loss of pathway function. The related family members, Vangl1, Celsr2 and Celsr3 (Formstone and Little, 2001; Doudney et al., 2005), might offer some functional redundancy in these mutants. We also cannot exclude the possibility that amelioration of the triple-mutant phenotype occurs owing to the presence of ‘protective’ genetic modifier alleles, unavoidably selected for in order to obtain viable double heterozygotes prior to breeding the triple mutants.

### Association of craniorachischisis with body wall defects

We observed a consistent association of craniorachischisis with abdominal wall closure defects, and a particularly increased abdominal wall defect penetrance in double heterozygotes containing the *Scrib^Crc^* mutant. Hence, 34% of *Vangl2*^*Lp*/+^;*Scrib*^*Crc*/+^ fetuses had abdominal wall defect compared with only 11% of *Vangl2*^*Lp*/+^;*Celsr1*^*Crsh*/+^ fetuses. Indeed, *Scrib^Crc/Crc^* mutants exhibited almost complete penetrance of abdominal wall defect (98%, *n*=50/51 at E14.5 or older), whereas less than 10% of *Celsr1^Crsh/Crsh^* and less than 5% of *Vangl2^Lp/Lp^* homozygotes had this defect. Despite both craniorachischisis and abdominal wall defect being morphogenetic closure defects, it is clear that they are not interdependent, and that the *Scrib* gene in particular is crucial for body wall closure. The mechanism of this dependence on Scrib remains to be determined.

## MATERIALS AND METHODS

### Mouse strains and genotyping

All animals were kept in accordance with the Animals (Scientific Procedures) Act 1986 of the UK Government, and with the Medical Research Council guidance in ‘Responsibility in the Use of Animals for Medical Research’ (July 1993).

*Circletail* (*Scrib^Crc^*) mutant mice arose as described previously (Murdoch et al., 2001b) and were initially maintained by random breeding between wild-type and heterozygotes within the colony. Subsequently, *Scrib*^*Crc*/+^ animals were outcrossed to C3H/HeH for six or more generations to create the subcongenic strain *Scrib^Crc^*-C3H. *Scrib^Crc^* mice carry a single-base insertion (3182–3183insC) within codon 947 of *Scrib* (Murdoch et al., 2003). Mice were genotyped using informative SNPs at 76.35 Mb and 74.85 Mb, closely flanking *Scrib* at position 76.05–76.07 Mb on Chr. 15 (positions from Ensembl release 71, April 2013). Primers for 15_76Mb SNP: Forward 5′Biotin-GACAGTGGGCAAGGCTGACA-3′, Reverse 5′-NNNGGCTGCACTTGTCGCTCAGA-3′, Sequencing (reverse) 5′-GCTCAGAGGACTCTCATC-3′. Primers for 15_74Mb SNP: Forward 5′-NNNCATTGGAAAACATGGGGAGGA-3′, Reverse 5′Biotin-AGCATCAGGGACAGGCAAGG-3′, Sequencing (forward) 5′-AAAACATGGGGAGGAC-3′. Pyrosequencing detects wild-type (A) or mutant (C) alleles for SNP15_76, and wild-type (C) or mutant (T) alleles for SNP15_74.

*Loop-tail* (*Vangl2^Lp^*) mice were obtained as described (Stanier et al., 1995) and used initially as F1 animals after breeding to SWR (Murdoch et al., 2001b) or CBA. Subsequently, *Vangl2*^*Lp*/+^ mice with a looped tail were bred to C3H/HeH for at least six generations, to create the subcongenic strain LPT-C3. *Vangl2^Lp^* mice carry a single-base substitution in *Vangl2*, c.1841G>A, creating a single amino acid change, S464N (Murdoch et al., 2001a). Embryos were genotyped for the mutation by pyrosequencing. Primers: Forward 5′Biotin-GTCCTGGCGCTTCAAGAGGA-3′, Reverse 5′-NNNGGCCAAACAGTGGACCTTGG-3′, Sequencing 5′-CAGTGGACCTTGGTGA-3′.

*Crash* (*Celsr1^Crsh^*) mice arose from ENU mutagenesis (Curtin et al., 2003) and were maintained initially as a congenic strain on the BALB/c background. Subsequently, *Celsr1*^*Crsh*/+^ mice with shaky-head behavior were bred to C3H/HeH for at least six generations, to create the subcongenic line *Celsr1^Crsh^*–C3. The *Celsr1^Crsh^* mutant carries a single-base substitution within *Celsr1*, c.3126A>G, causing the amino acid change D1040G (Curtin et al., 2003). Mice were genotyped for this mutation by pyrosequencing. Primers: Forward 5′Biotin-AGCCCTGTGGGTTCAGTGGT-3′, Reverse 5′-NNNGGAAGACCTCGGGCACATTG-3′, Sequencing 5′-ACCTTCGTCCGGG-3′.

*Spin-cycle* (*Celsr1^Scy^*) mice arose from ENU mutagenesis (Curtin et al., 2003) and were maintained as a congenic strain on the C3H/HeH background. *Celsr1^Scy^* mutants carry a single-base substitution within Celsr1, c.3337T>A, causing the amino acid change N1110K (Curtin et al., 2003). Mice were genotyped for this mutation by pyrosequencing. Primers: Forward 5′-AATGTGCCCGAGGTCTTC-3′, Reverse 5′Biotin-GGGAAGCTGTTGGATTTATTG-3′, Sequencing 5′-CGTCTCCTGGACCAGAA-3′.

Mice were genotyped from ear notch samples, and embryos from yolk sac. Tissues were digested in proteinase K for 3–6 hours. Digests were used as PCR templates, with 20-μl reactions containing 1× reaction buffer with 1.5 mM magnesium chloride, 0.2 mM each dNTP, 0.4 μM forward and reverse primers, 0.7 units Thermoprime Polymerase (Abgene). Products were amplified using a thermocycler (MJ Research) with 95°C for 5 minutes, then 30–40 cycles of: 95°C 15 seconds, 55–65°C 30 seconds, 72°C 30 seconds. Reactions were purified using streptavidin beads and analyzed by pyrosequencing (Qiagen).

### Embryo generation and analysis of neural tube closure

Mice were kept in a controlled 12-hour light/12-hour dark cycle, where the light cycle began at 7 am. Embryos were generated by timed mating between heterozygous animals, with noon on the day of finding a vaginal plug designated E0.5. To obtain E8.0 stage embryos, mice were housed using a reversed light/dark cycle, with the light cycle beginning at 10 pm. Embryos were dissected from the uterus in phosphate buffered saline (PBS) containing 10% newborn calf serum (Invitrogen), examined and photographed on a Leica MZ16 stereomicroscope. Embryos were scored as achieving Closure 1 when there was evidence of contact between the neural folds in the mid-region of the embryo, such that the neural folds could no longer be distinguished and a continuous surface ectoderm was observed.

### Protein extraction and western blotting

Total cell lysates were generated by homogenization in ice-cold RIPA buffer: PBS plus 1% Nonidet P40, 0.1% SDS, 0.5% sodium deoxycholate and protease inhibitor cocktail for mammalian tissues (Sigma P8340). Lysates were incubated on ice for 30 minutes, then centrifuged to remove insoluble material. Membrane and soluble protein fractions were obtained using the Proteoextract Native Membrane extraction kit (Calbiochem). Extracts were obtained from single embryos at E10.5 or older, or from pools of five embryos at E8.5. Experiments were repeated at least three times, using independent protein samples. Aliquots of protein extracts were stored at −70°C. For dephosphorylation experiments, protein samples were prepared using the ProteoExtract Subcellular Fractionation kit (Calbiochem); fraction 2 (membranes and organelles) was incubated with shrimp alkaline phosphatase (Promega) at 37°C.

Protein samples were electrophoresed through 7% or 3–8% NuPAGE Tris-acetate gels (Invitrogen), with 1 μg per lane, then electroblotted onto Hybond ECL nitrocellulose (GE Healthcare). Blots were blocked overnight at 4°C in 2% (w/v) ECL Advance blocking agent (GE Healthcare) in TBST (137 mM NaCl, 200 mM Tris-HCl pH 7.6, 0.1% Tween-20), incubated with primary antibody in blocking solution (1 hour, room temperature), washed in TBST, incubated with secondary antibody in blocking solution (1 hour) then developed with ECL Advance (GE Healthcare). Bands were quantified from scans of film after short exposures, using Adobe Photoshop, and normalized against the intensity of β-tubulin or fatty acid synthase bands. Significance was determined with a paired Student’s *t*-test using the normalized values. Primary antibodies: goat anti-Vangl2 (N-13) (1:1000, sc- 46561, Santa Cruz), mouse anti-Scrib [1:500, (Dow et al., 2003)], rabbit anti-Celsr1 [1:3000 (Formstone et al., 2010)], rabbit anti-β-tubulin (1:1000, sc-9104, Santa Cruz) and mouse anti-fatty acid synthase (1:1000, sc-55580, Santa Cruz). Secondary antibodies were horseradish peroxidase (HRP)-conjugated anti-mouse, goat anti-rabbit or rabbit anti-goat antibodies (1:10,000, DAKO).

## Supplementary Material

Supplementary Material
