# Supplementary material for: Genetic interactions between planar cell polarity genes cause diverse neural tube defects in mice
Source: Dis Model Mech. 2014 Aug 15;7(10):1153–63. doi: 10.1242/dmm.016758 (PMC4174526; doi:10.1242/dmm.016758)
Supplement: Supplementary Material [file supp_7_10_1153__index.html]

Supplementary Material 

# Genetic interactions between planar cell polarity genes cause diverse neural tube defects in mice

## DMM016758 Supplementary Material

**Files in this Data Supplement:**

- **Supplementary Material**
