# Supplementary material for: Genetic interactions between planar cell polarity genes cause diverse neural tube defects in mice
Source: Dis Model Mech. 2014 Aug 15;7(10):1153–63. doi: 10.1242/dmm.016758 (PMC4174526; doi:10.1242/dmm.016758)
Supplement: Supplementary Material [file supp_016758_DMM016758.pdf]

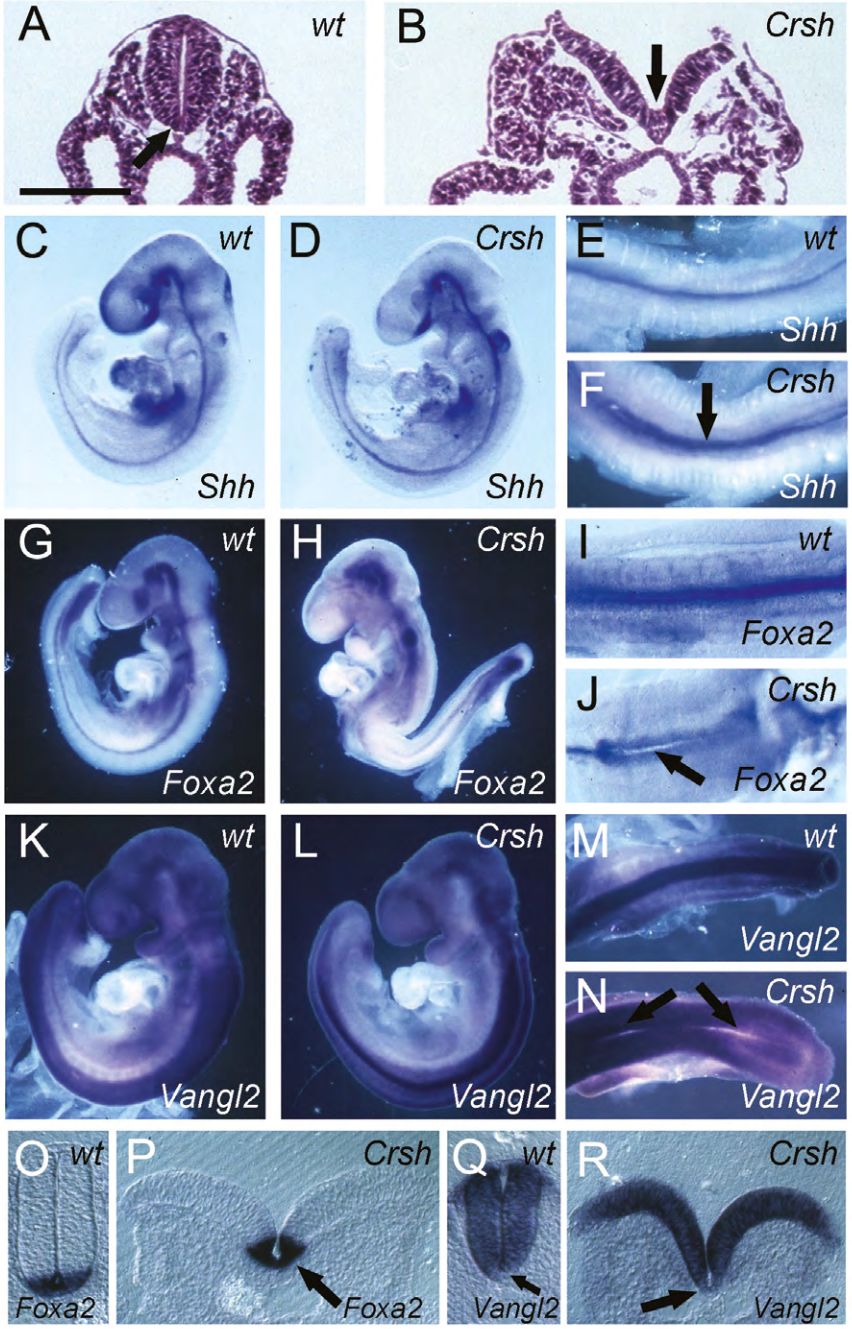

Supplementary Fig. 1. *Celsr1$^{Crsh/Crsh}$* mutants exhibit midline abnormalities of the neuroepithelium at the stage of neural tube closure initiation. (A,B) H & E stained transverse sections through E8.5 (10 somite stage) embryos, just after the stage of closure initiation. The wild-type embryo (A) shows a compact ventral midline in the neuroepithelium (A, arrow), whereas the *Celsr1$^{Crsh/Crsh}$* homozygous mutant littermate exhibits a persistently open neural tube with enlarged ventral midline region (B, arrow). (C-J) *In situ* hybridisation for the midline markers *Shh* (C-F) and *Foxa2* (G-J) reveal similar expression patterns in wild-type (C,G) and *Celsr1$^{Crsh/Crsh}$* mutants (D,H) when viewed laterally, but dorsal views of the caudal neural tube reveal an enlarged or bifurcated midline in the mutant (arrows in F,J) compared with wild-type (E,I). (K-N) *In situ* hybridisation for *Vangl2* reveals similar expression in wild-type (K) and *Celsr1$^{Crsh/Crsh}$* homozygous (L) embryos when viewed laterally (K,L), but dorsal views of the caudal neural tube reveal a widened ventral midline that is negative for *Vangl2* expression in the mutant (arrows in N), although not in wild-type (M). (O,P) Transverse sections of embryos hybridized as whole mounts for *Foxa2* expression. Compared with wild-type (O), the *Celsr1$^{Crsh/Crsh}$* embryo (P) exhibits a widened domain of *Foxa2* expression. (Q,R) Transverse sections of embryos hybridized as whole mounts for *Vangl2* expression. Similar expression patterns are seen in wild-type (Q) and *Celsr1$^{Crsh/Crsh}$* (R), although the ventral midline region that lacks *Vangl2* expression (R, arrow) appears wider in the *Celsr1$^{Crsh/Crsh}$* mutant. Scale bar in (A) represents 0.1 mm (A,B), 1.25 mm (C,D,G,H), 0.6 mm (E,F,M-P), 0.5 mm (I,J), 1 mm (K,L), 0.16 mm (Q,R).

Supplementary Table 1. Genotype distribution of offspring from intercrosses between *Vangl2*$^{Lp/+}$, *Scrib*$^{Crc/+}$, *Celsr1*$^{Crsh/+}$ and *Celsr1*$^{Scy/+}$ on C3H/HeH background.

Cross (female x male)	Genotype [a]				Total	Ratios [b]
Scrib$^{Crc/+}$ **x** *Vangl2*$^{Lp/+}$	*Lp/+; Crc/+*	*Lp/+; +/+*	*+/+; Crc/+*	*+/+; +/+*		*Lp*:+ = 66:42
Observed no.	32	34	26	16	108	*Crc*:+ = 58:50
Expected no.	27	27	27	27		$p < 0.05$ [c]
Vangl2$^{Lp/+}$ **x** *Celsr1*$^{Crsh/+}$	*Lp/+; Crsh/+*	*Lp/+; +/+*	*+/+; Crsh/+*	*+/+; +/+*		*Lp*:+ = 41:32
Observed no.	25	16	13	19	73	*Crsh*:+ = 38:35
Expected no.	18.25	18.25	18.25	18.25		$p > 0.05$
Scrib$^{Crc/+}$**x** *Celsr1*$^{Crsh/+}$	*Crc/+; Crsh/+*	*Crc/+; +/+*	*+/+; Crsh/+*	*+/+; +/+*		*Crc*:+ = 82:89
Observed no.	41	41	49	40	171	*Crsh*:+ = 90:81
Expected no.	42.75	42.75	42.75	42.75		$p > 0.05$
Celsr1$^{Scy/+}$**x** *Scrib*$^{Crc/+}$	*Crc/+; Scy/+*	*Crc/+; +/+*	*+/+; Scy/+*	*+/+; +/+*		*Crc*:+ = 28:28
Observed no.	18	10	16	12	56	*Scy*:+ = 34:22
Expected no.	14	14	14	14		$p > 0.05$

[a] Genotype of offspring collected from each intercross.

[b] Ratio of mutant: wild-type allele for each gene.

[C] χ^2 tests to assess whether observed ratios of mutant alleles in offspring differ statistically from expected values.

Supplementary Table 2. Penetrance of heterozygote phenotypes before and after generation of sub-congenic strains on C3H/He background.

	Phenotype	Penetrance in offspring of heterozygote crosses [1]		Statistical comparison
		Original background	C3H/He background	
Celsr1$^{Crsh/+}$	Shaky-head behaviour [2]	275/811 (68%)	68/425 (32%)	$p < 0.001$
Vangl2$^{Lp/+}$	Looped tail [3]	614/1361 (90%)	226/818 (55%)	$p < 0.001$

[1] Penetrance calculated on the basis that half of the offspring would be expected to exhibit the defect, if fully penetrant.

[2] Shaky-head behaviour is characteristic of *Celsr1*^{Crsh} heterozygotes, and likely derives from vestibular dysfunction.

[3] The looped tail defect characterizes *Vangl2*$^{Lp/+}$ heterozygotes and reflects delayed closure of the spinal neural tube (Copp et al., 1994). *Vangl2*$^{Lp/+}$ tail defects were less severe on the C3H/HeH background, with frequent occurrence of a loose loop or kink instead of the tight looping or knot that is more characteristic of the phenotype on the original LPT/CBA background.

Supplementary Table 3. Intercrosses between *Celsr1*$^{Crsh/+}$ females and *Vangl2*$^{Lp/+}$*; Scrib*$^{Crc/+}$ males generate all classes of offspring and show a range of phenotypes

	Genotype at each locus								Ratios[a]
***Vangl2* (*Lp* locus)**	*Lp/+*	*Lp/+*	*Lp/+*	*Lp/+*	+/+	+/+	+/+	+/+	12:10
***Scrib* (*Crc* locus)**	*Crc/+*	*Crc/+*	+/+	+/+	*Crc/+*	*Crc/+*	+/+	+/+	13:9
***Celsr1* (*Crsh* locus)**	*Crsh/+*	+/+	*Crsh/+*	+/+	*Crsh/+*	+/+	*Crsh/+*	+/+	13:9
**Number**	3	4	3	2	4	2	3	1	
**Phenotypes[b]**	CRN	LT	CRN	LT	Normal	Normal	LT	Normal	
	CRN	LT	CRN	LT	Normal	Normal	Normal		
	EX	LT	LT		Normal		Normal		
		LT			LT				

[a] Ratio of mutant: wild-type alleles observed at each locus.

[b] Phenotypes of individual fetuses: CRN, craniorachischisis; EX, hindbrain exencephaly; LT, looped tail.
